# Maternal antidepressant effects on the fetal nonstress test

**DOI:** 10.1007/s00737-025-01607-9

**Published:** 2025-07-03

**Authors:** Emily McCauley, Alyssa Thompson, Samantha Gawrys, Jason Benedict, Jonathan Schaffir

**Affiliations:** 1 The Ohio State University College of Medicine, Columbus, OH USA; 2 Center for Biostatistics, The Ohio State University, Columbus, OH USA; 3 Wexner Medical Center Department of Obstetrics and Gynecology, The Ohio State University, Columbus, OH USA

**Keywords:** Fetal assessment, Nonstress test, Antidepressants, Selective serotonin reuptake inhibitors

## Abstract

**Purpose:**

Selective serotonin reuptake inhibitors (SSRIs) are commonly prescribed for mood disorders in pregnancy, though studies regarding effects on fetal behavior are conflicting. Previous studies have suggested that fetuses exposed to these medications in the third trimester may have decreased fetal heart rate variability. Since changes in variability may affect the interpretation of antenatal testing, clinicians should be aware of those medications that may impair effective testing.

**Methods:**

A retrospective observational cohort study was performed to compare nonstress test (NST) parameters of fetuses exposed to SSRIs with unexposed fetuses at 36 weeks gestation. Subjects were excluded if they had multiple gestations, fetal anomalies including growth restriction, illicit substance use or use of other psychotropic medications. NSTs were compared for fetal baseline heart rate, variability, time to reactivity, number of accelerations over time, and number of fetal movements over time.

**Results:**

Of 219 participants, 12 were taking an SSRI/SNRI at the time of their 36-week NST. There were no significant differences in demographics or indications for NST between groups. NSTs were reactive in 92% of those taking an SSRI/SNRI and in 97% of those not taking an SSRI/SNRI. Time to reactivity [8.6 min vs. 12.5 min], baseline heart rate [142 bpm vs. 139 bpm], and heart rate variability [12.1 bpm vs. 11.7 bpm] were all similar between the two groups.

**Conclusion:**

Though limited by sample size, lack of pharmacologic data and potential confounding by indication, the study suggests SSRI use is not associated with any significant changes in NST. Clinicians should be reassured that NST assessment remains a useful means of determining fetal wellbeing in patients using these medications.

## Article Highlights

• SSRIs are commonly used in pregnancy, but may have effects on fetal movement and heart rate characteristics

• Since nonstress testing relies on these measurements, interpretation may be confounded in patients using these medications

• This study compared nonstress tests in fetuses exposed or not exposed to these medications

• No difference was found in NST characteristics, suggesting unlikely impact on NST interpretation

## Introduction

Mental health disorders are some of the most common comorbid conditions during pregnancy, affecting between 9 and 20 percent of pregnancies (American College of Obstetricians and Gynecologists 2023; Bruce et al 2008), and are often treated with psychotropic medications such as selective-serotonin reuptake inhibitors (SSRI) and serotonin and norepinephrine reuptake inhibitors (SNRI). SSRI/SNRIs have been clinically embraced as safe and effective treatments for perinatal mood and anxiety disorders (PMAD) (Raffi et al 2019). However, there is some data to suggest that this class of medications (Emory and Dieter 2006) or depression itself (Dieter et al 2008) may impact fetal blood flow, with increased middle cerebral artery velocity in fetuses exposed to SSRIs, and activity levels, which are increased in mid-pregnancy for fetuses exposed to maternal depression. As clinicians rely on such parameters to assess fetal well-being, it is important to determine whether these exposures may affect interpretation of assessment tools such as the fetal nonstress test (NST).

The NST is one of the most frequently used techniques for assessing fetal wellbeing during pregnancy. The goal of antepartum testing, such as the NST, is to reduce the rate of intrauterine fetal demise by identifying fetuses at risk of death and intervening promptly to avoid this possibility (Greenberg and Druzin 2021). The late third trimester is the most common time period when this testing is initiated, when the fetus is most at risk for compromised blood flow but mature enough to thrive if delivery is indicated. Common indications where this risk exists include pre-existing maternal conditions (e.g. diabetes mellitus or hypertension), placental conditions (e.g. post-datism or single umbilical artery), or fetal conditions (e.g. growth restriction) (Umana and Siccardi 2020). Its advantages are wide availability in the outpatient setting and non-invasive assessment. As a non-invasive test, it relies on indirect assessment of fetal health by using fetal movements and heart rate characteristics such as baseline, variability, and accelerations as indicators of an intact and mature nervous system, suggesting a healthy, well-oxygenated fetus.

Because these parameters are surrogate indicators for fetal wellbeing, a test with an inadequate number or degree of heart rate accelerations (referred to as “non-reactive”) has a high false-positive rate ranging from 50–90% in various studies (Greenberg and Druzin 2021). Situations leading to false-positive results may require intervention resulting in costly additional tests, morbidity from premature delivery, and maternal anxiety. It is, therefore, important to identify common extrinsic effects on fetal behavior that might negatively impact fetal movement and heart rate characteristics even in the presence of normal oxygenation and perfusion.

Maternal mood is one such issue which may affect fetal activity. Maternal mood has been associated with changes in fetal heart rate variability, with lower variability demonstrated in depressed women using SSRIs (Campbell et al 2021, 2024) and in women with increased depressive symptoms in the third trimester (Pinto et al 2023). However, a study specifically examining associations between symptoms of PMAD and interpretation of the fetal NST showed no changes in any NST parameters with depressed mood (McCauley et al 2023).

Maternal medication is another such issue, with some substances clearly having an impact on NST interpretation. For example, opiates and other depressants have been demonstrated to negatively impact the results of antepartum testing (Kopel and Hill 2013). Methamphetamines have been implicated in affecting intrauterine behavior, though they have not been shown to affect antepartum testing (Salisbury et al 2009). SSRIs are another class of medication that could conceivably affect fetal behavior, as these compounds may affect serotonin activity in the fetal brain (Brummelte et al 2017), or have an effect peripherally on the autonomic nervous system. A study comparing exposed and non-exposed fetuses demonstrated increased fetal movement in mid-pregnancy for patients using SSRI but not in late pregnancy (Gustafsson et al 2018), and another study demonstrated an increase in the proportion of jerky movements in fetuses exposed to SSRI in late pregnancy (Salisbury et al 2009). In a study of depressed pregnant women in the third trimester, there was decreased fetal heart rate variability and fewer fetal heart rate accelerations in patients treated with SSRIs compared with those who were not exposed to the medication (Rurak et al 2011)_._ A subsequent study by the same group using a larger sample demonstrated only the decreased variability in exposed fetuses (Campbell et al 2021). Other studies have suggested a dose effect, with increases in number of fetal heart rate decelerations (Campbell et al 2021) or increased motor activity (Mulder et al 2011) with higher SSRI doses.

Although associations between SSRIs and fetal characteristics have been investigated, there have not been any studies to date that examine interpretation of clinically indicated NSTs in a typical clinical setting in patients using these medications. Due to the potential fetal impact of SSRI use, NSTs of pregnant persons using SSRIs may be interpreted differently as compared to pregnant persons not taking SSRIs. However, we hypothesize that the impact is likely to be small enough so as not to change test interpretation. In those studies cited above, the differences were statistically significant but not likely to be clinically significant, as with Rurak et al., where the number of accelerations differed from 14 to 16 over 50 min, or Mulder et al., where fetal movements differed from 12 to 15 over one hour. These numbers would still be ample to meet criteria for reactivity on the NST. The aim of the study is to compare NST parameters and interpretation between individuals using SSRI/SNRI and those who are not to see if there are associations that may warrant alternative testing considerations in this population.

## Materials and methods

The records of all pregnant patients undergoing NSTs at a single academic medical center prenatal clinic were reviewed over the course of one year. The clinic is located in a midwestern American city, serving a range of high- and low-risk pregnant patients of low income. Data from the electronic medical record of all individuals who had a 36-week NST at the clinic between 1/1/2022–12/24/2022 were examined. With past clinic volume demonstrating approximately 400 patients receiving NST in one calendar year, we anticipated that this interval would be adequate for recruiting an appropriate sample size. Exclusion criteria included multiple gestation, fetal anomaly including fetal growth restriction (FGR), opiate use disorder, or use of other psychotropic medication. This class of medication was excluded to eliminate confounding influences of other substances that may affect mood and anxiety. Substance use was abstracted from documentation in the electronic medical record (EMR); urine drug screening was not referenced nor routinely available. Demographic information was collected including age, race/ethnicity, gravidity, parity, active or previous mood disorder diagnosis, prescribed medications (specifically SSRI/SNRI use and type), fetal sex, and indication for NST. Mood disorder diagnoses and medication use had been recorded in the EMR by patient report.

The NST was analyzed for reactivity (defined as two 15-s accelerations of 15 beats per minute above the baseline within 20 min), baseline heart rate, variability of heart rate, frequency of accelerations, time to reactivity, recorded fetal movements and use of vibroacoustic stimulation. Fetal movements were indicated by the patient with use of a self-activated button. Baseline and variability were defined by currently accepted obstetrical parameters (Macones et al 2008). Variability was defined as the difference between maximum and minimum recorded heart rate over one minute of baseline (absent accelerations) recording. Vibroacoustic stimulation was provided at the discretion of the administering nurse when the fetus was thought to be in a quiescent state; no standard length of test was anticipated, but nurses would often provide stimulation if they anticipated a test longer than 30 min based on lack of fetal activity. 36 weeks was chosen as a standardized point in pregnancy when NSTs are commonly initiated, and criteria for reactivity in a healthy fetus should be met. Those who had multiple NSTs during the 36th week of pregnancy had only the first NST examined. NSTs were reviewed retrospectively and analyzed by one of three researchers with equivalent training in interpreting the tests who were blinded to medication exposure. NSTs and chart information results were compared between those using SSRI/SNRIs at the time of the test and those who were not.

As previous literature cited above (Campbell et al 2024, Pinto et al. 2023) has suggested that the greatest impact of SSRI/SNRI medication may be on fetal heart rate variability, change in this parameter was the primary outcome of interest. With approximately 1 in 10 clinic patients in this population having previously reported using an SSRI during pregnancy (Lendel et al 2024), we planned to compare data in a 1:10 ratio. To demonstrate a 25% decrease in mean variability with 80% power at a significance level of 0.05 (using a mean variability of 9 beats per minute and a standard deviation of 3, as determined by a previous study at this institution [(McCauley et al 2023)], we calculated a sample size of 209 patients (19 on SSRI and 190 unexposed) needing to be included in the study.

Variables of interest were analyzed with the mean and standard deviation for continuous variables and the number and percentage for categorical variables presented. All outcomes of interest were assessed using either Fisher’s exact test for categorical variables or two-sample t-tests for continuous variables. *P*-values < 0.05 were considered statistically significant, and all *p*-values are presented at their nominal levels as this study is exploratory in nature. To ensure the results were robust to potential outliers, Wilcoxon rank-sum tests were used to assess uniformity of results compared to t-tests with all continuous variables. All analyses were conducted in R version 4.4 (R Core Team (2024). R: A Language and Environment for Statistical Computing. R Foundation for Statistical Computing, Vienna, Austria. < https://www.R-project.org/ >).

The study was approved by the Institutional Review Board of the Ohio State University. Because data was collected retrospectively, informed consent was not deemed necessary.

## Results

There were 360 potential participants who had an NST at this clinic between 1/1/2022 and 12/24/2022. 141 subjects were excluded, either due to absence of a 36-week NST (*n = *70), multiple gestation (*n = *7), FGR/fetal anomaly (*n = *41), substance use (including opiates, cocaine, methamphetamine, bath salts) (*n = *20), or other psychotropic medication use (including aripiprazole, clozapine, and quetiapine) (*n = *3). 219 records met eligibility criteria, 12 (5.5%) of whom were taking SSRI/SNRI at the time of the 36-week NST.

Demographics between the groups were similar (Table 1), other than an expected difference in prior diagnosis of mood disorder. However, comparisons of race were limited because 1/3 of respondents did not report identification with one of the races listed. The percentage of individuals identifying as Hispanic was 35% in the group without antidepressants compared to 8% in the SSRI/SNRI users, but the difference was not statistically significant.

**Table 1:** Demographics

Demographics	SSRI negative (*n = *207)	SSRI positive (*n = *12)	*p* value
Age (in years), mean (SD)	32.0 (6.2)	33.2 (4.1)	0.37
Race:			0.06
Black	78 (37.7)	6 (50.0)	
White	40 (19.3)	5 (42.7)	
Asian or South Asian/Indian	11 (5.3)	0 (0.0)	
2 or more races	7 (3.4)	1 (8.3)	
Other	70 (33.8)	0 (0.0)	
Refused to answer	1 (0.5)	0 (0.0)	
Ethnicity:			
Non-Hispanic	132 (63.8)	11 (91.7)	0.06
Hispanic	72 (34.8)	1 (8.3)	
Refused to answer	1 (0.5)	0 (0.0)	
Gravidity, mean (SD)	3.7 (2.0)	4.4 (2.1)	0.24
Parity, mean (SD)	1.9 (1.4)	2.4 (1.6)	0.23
Mood disorder diagnosis	34 (16.4)	12 (100.0)	< 0.0001
Fetal sex			0.99
Male	103 (49.8)	6 (50.0)	
Female	104 (50.2)	6 (50.0)	
Indication for test:			*
Diabetes	99 (48%)	5 (42%)	
Hypertension	35 (17%)	3 (25%)	
Advanced maternal age	63 (31%)	4 (33%)	
Other maternal disease	64 (31%)	2 (17%)	

^1^Presented as *n* (%) unless otherwise noted

*No *p*-value provided as more than 1 may be present

In the group unexposed to SSRI/SNRI, 16% of participants had a previous/current mood disorder diagnosis but were not taking an antidepressant at the time of the NST, while 100% of SSRI/SNRI users had a current or previous mood disorder diagnosis. The most common indications for undergoing NST were diabetes (*n = *104), advanced maternal age (*n = *67), and hypertension (*n = *38). 68 participants had a variety of other maternal conditions, including class 3 obesity. There were no differences in indications between the two groups.

Of the participants taking an SSRI/SNRI (*n = *12), medications represented included citalopram (8.3%, *n = *1, prescribed as 20 mg daily), escitalopram (25%, *n = *3; 1 prescribed 10 mg daily and 2 prescribed 20 mg daily), fluoxetine (25%, *n = *3, 1 prescribed 10 mg daily, 1 prescribed 20 mg daily, and 1 prescribed 40 mg daily), sertraline (33%, *n = *4; 3 prescribed 50 mg daily and 1 prescribed 100 mg daily), and venlafaxine (8.3%, *n = *1, 75 mg daily).

**Fig. 1:**
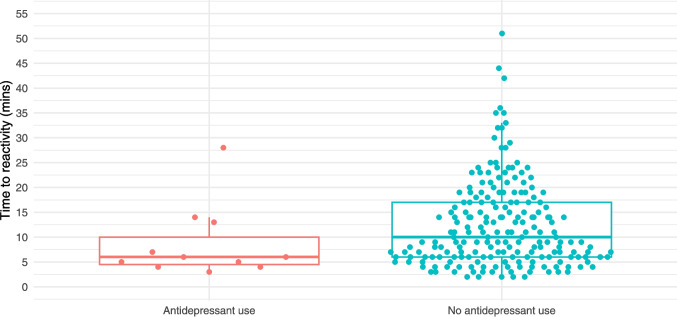
Scatter box plot of time to NST reactivity

**Fig. 2:**
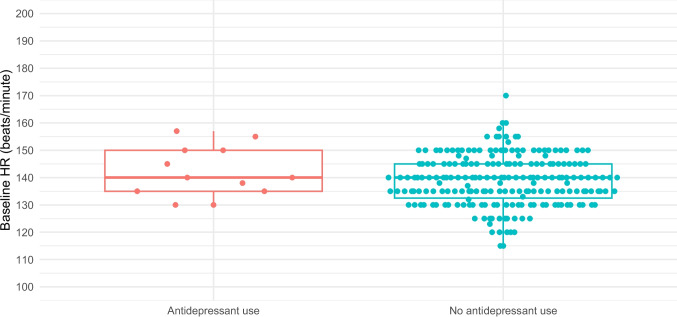
Scatter box plot of baseline fetal heart rate

**Fig. 3:**
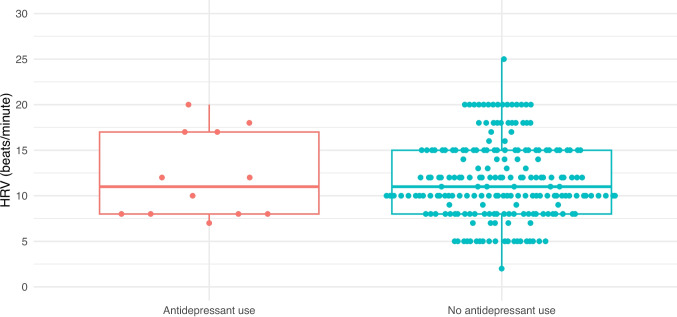
Scatter box plot of heart rate variability

**Fig. 4:**
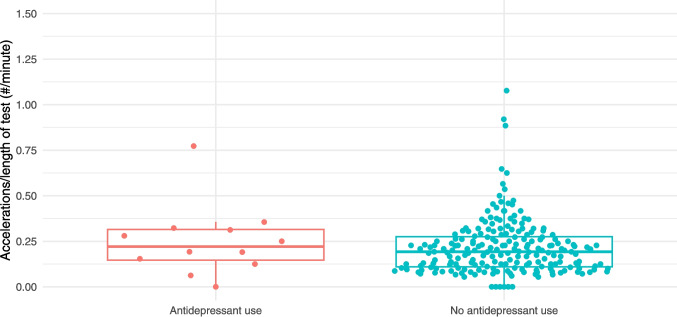
Scatter box plot of heart rate accelerations over length of NST

**Fig. 5:**
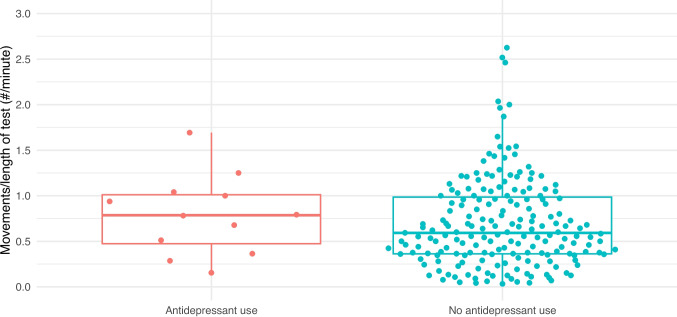
Scatter box plot of fetal movements over length of NST

NSTs were reactive in 97% (200/207) of unexposed patients and 92% (11/12) of those taking an SSRI/SNRI. As demonstrated in Table 2, time to reactivity, baseline heart rate, heart rate variability, and rate of vibroacoustic simulation were all similar between the two groups. Figures 1, 2, 3, 4, and 5 provide scatter box plots that illustrate the similarities between groups on each analysis. There were no significant differences between the two groups in recorded fetal movements or number of accelerations divided by the length of the test.

**Table 2:** NST Results for entire group

NST^1^	SSRI negative (*n = *207)	SSRI positive (*n = *12)	*P*-value
Reactive	201 (97.1%)	11 (91.7%)	0.33
Time to reactivity (mins)	12.5 (8.7)	8.6 (7.4)	0.12
Baseline heartrate (bpm)	139 (9.2)	142.1 (9.2)	0.28
Heart rate variability (bpm)	11.7 (4.3)	12.1 (4.7)	0.77
Length of test (mins)	28.3 (9.9)	30.4 (9.4)	0.29
Number of accelerations/length of test	0.2 (0.2)	0.24 (0.2)	0.55
Recorded fetal movements/length of test	0.67 (0.7)	0.74 (0.6)	0.73
Use of VAS	56 (28%)	2 (18%)	0.73

^1^Presented as mean (SD) for continuous variables and n (%) for categorical variables

*bpm* beats per minute, *VAS* vibroacoustic stimulation

To improve confidence that this null finding reflects the use of antidepressants and not a history of mood disorders, a separate analysis was performed to compare the antidepressant group with those participants with a history of mood disorder but no medication use (Table 3). No significant differences were noted; however the group sizes are small, so the similarity could signify either the absence of an effect or a lack of statistical power to detect differences.

**Table 3:** NST Results for those with prior diagnosis of mood disorder

NST^1^	SSRI negative (*n = *34)	SSRI positive (*n = *12)	*P*-value
Reactive	32 (94%)	11 (91.7%)	0.30
Time to reactivity (mins)	13.3 (8.0)	8.6 (7.4)	0.08
Baseline heartrate (bpm)	139.7 (9.5)	142.1 (9.2)	0.45
Heart rate variability (bpm)	11.1 (4.0)	12.1 (4.7)	0.48
Number of accelerations/length of test	0.17 (0.1)	0.24 (0.2)	0.12
Recorded fetal movements/length of test	0.73 (0.5)	0.74 (0.6)	0.95
Use of VAS	9 (27%)	2 (18%)	0.70

^1^Presented as mean (SD) for continuous variables and n (%) for categorical variables

*bpm* beats per minute, *VAS* vibroacoustic stimulation

## Discussion and conclusions

In this retrospective analysis, fetuses exposed to SSRI/SNRI at 36 weeks had NST findings similar to those of the unexposed cohort. Although the number of antidepressant users in the study was small and the effect of the medication could not be distinguished for certain from the indication for use, the results suggest that prenatal care providers may use similar criteria in interpreting NSTs for patients who do or do not use these medications.

In early pregnancy, a common topic of discussion between patients and their providers is how to effectively treat mental health disorders in a way that is safe for both the fetus and the parent. For appropriately counseled individuals, antidepressant use during pregnancy is recommended in the setting of PMAD as the risk associated with untreated maternal depression/anxiety, particularly when moderate to severe, may be higher than the potential risk of SSRI/SNRI use on the fetus (Susser et al 2016). Our results help to reassure obstetric providers that these medications are unlikely to affect fetal monitoring in late pregnancy. Although it is possible that depressed mood itself can impact fetal heart rate variability (Semeia et al 2023), our results suggest limited effect on interpreting this particular test in women treated for mood disorders.

Strengths of the study included a uniform cohort at a single gestational age and consistency in NST analysis. We excluded subjects for factors that were more likely to suppress heart rate variability or increase the likelihood of a nonreactive test such as substance use or fetal anomaly, which makes the findings less likely to be confounded by other issues. Additionally, NST interpretation was carried out by three individuals with standardized training according to established guidelines and blinded to medication exposure. Although the study sample was fairly homogeneous, this clinic’s clientele is predominantly urban and low-income, so the results may not be generalizable to all prenatal clinics.

A limitation of our study was the lack of reliable information related to dosing or timing of medication prior to the fetal testing. Maternal self-report may not reflect true drug exposure status, and inaccurate reporting may impact the findings on this small sample. Prior studies have suggested that fetal activity changes may be seen at peak levels after drug administration, but not when levels were low (Campbell et al 2021). In our study, the possibility of low serum levels due to non-adherence to prescribed regimens and possible long intervals from administration may have contributed to the negative findings. Without knowing when participants were tested relative to taking medication, or what serum level they achieved, the effect of these medications on the NST remain uncertain. Additional research measuring serum levels would be needed to answer this question. However, our results reflect what could more practically be expected in clinical practice, since doses used by subjects were typical of those given in pregnancy and testing was not timed to coincide with dosing.

Although we saw no difference in results between participants with a history of mood disorder who were or were not prescribed antidepressant medication, the study could not definitively eliminate potential confounding by indication. In this sample, the effects of exposure to medication could not be fully disentangled from the underlying mental health condition. Our study also lacked information on participant mood at the time of the assessment. Results may have been confounded by depressive symptoms in our participants, since some studies have suggested associations between fetal heart parameters and mood alone (Pinto et al 2023), and even those using medication may still have experienced depressed mood at the time of testing. We also lack data on the long-term treatment response of those patients who were taking antidepressants; even treated patients with poor response to pharmacotherapy may demonstrate changes in fetal parameters (Campbell et al 2021, 2024). However, the findings of a separate study showing no association between concurrent mood and NST characteristics in a similar sample support the inclusion of participants with history of mood disorder who were not prescribed antidepressants (McCauley et al 2023). Furthermore, additional analysis of unmedicated participants with a history of mood disorder in our study demonstrated no significant difference in the parameters evaluated. Still, further examination of antepartum testing data with respect to the mood response in treated and untreated patients is needed.

Another limitation of the study is the lack of statistical power due to a smaller-than-expected percentage of women using medication to treat depression at this point in pregnancy. The prevalence of SSRI/SNRI use at 36 weeks was considerably less than the 10% expected based on previous work in this population (Lendel et al 2024). This discrepancy between expected and actual prevalence of SSRI/SNRI use may reflect a decline in compliance with medication use over the course of pregnancy. However, there was no trend to suggest that a higher prevalence of SSRI use in this study sample would have significantly changed the results. The possibility of inter-rater variability in interpretation of the NSTs is also a limitation.

Our study was too small to examine the effects of specific indications for testing, specific medications, or whether other atypical antidepressants may have different effects. Future studies with greater numbers of individuals treated for depression may provide more information on each compound’s unique effects on fetal behavior. Given the possibility that women with depressed mood may have discontinued prescribed medication, we could not determine if untreated depression may have had an impact. In a study comparing SSRI-exposed fetuses in women with and without depressed mood, episodes of high heart rate variability decreased only in the non-depressed group (Campbell et al 2021). However, a prior study in a similar sample determined that acute mood changes do not affect NST interpretation (McCauley et al 2023). Future studies of NST interpretation that specifically examine the relations of anti-depressant exposure and mood at the time of testing would be informative.

This modest study’s conclusions are tempered by its limitations in sample size, information on participant mood and lack of pharmacologic data. Nevertheless as a practical study that specifically examines antidepressant effects on clinically indicated nonstress tests, this study is reassuring regarding NST as a reliable form of antepartum assessment for patients who choose to continue SSRI/SNRI use in the late third trimester. Larger studies with enough users to examine specific medications and compliance with dosing would provide even greater reassurance.

## Data Availability

Data from this study is protected health information and not available for public sharing.
